# In silico studies of the open form of human tissue transglutaminase

**DOI:** 10.1038/s41598-024-66348-8

**Published:** 2024-07-10

**Authors:** S. D. Ivashchenko, D. A. Shulga, V. D. Ivashchenko, E. V. Zinovev, A. V. Vlasov

**Affiliations:** 1 Moscow Institute of Physics and Technology, Dolgoprudny, Russia 141701; 2 Department of Chemistry, Moscow State University, Moscow, Russia 119991; 3 Laboratory of Microbiology, BIOTECH University, Moscow, Russia 125080; 4 Joint Institute for Nuclear Research, Dubna, Russia 141980

**Keywords:** Molecular modelling, Cheminformatics, Protein structure predictions, Virtual screening, Computational chemistry

## Abstract

Human tissue transglutaminase (tTG) is an intriguing multifunctional enzyme involved in various diseases, including celiac disease and neurological disorders. Although a number of tTG inhibitors have been developed, the molecular determinants governing ligand binding remain incomplete due to the lack of high-resolution structural data in the vicinity of its active site. In this study, we obtained the complete high-resolution model of tTG by in silico methods based on available PDB structures. We discovered significant differences in the active site architecture between our and known tTG models, revealing an additional loop which affects the ligand binding affinity. We assembled a library of new potential tTG inhibitors based on the obtained complete model of the enzyme. Our library substantially expands the spectrum of possible drug candidates targeting tTG and encompasses twelve molecular scaffolds, eleven of which are novel and exhibit higher binding affinity then already known ones, according to our in silico studies. The results of this study open new directions for structure-based drug design of tTG inhibitors, offering the complete protein model and suggesting a wide range of new compounds for further experimental validation.

## Introduction

Human tissue transglutaminase (tTG), also known as protein-glutamine gamma-glutamyltransferase 2 (UniProt ID:P21980), is a 79 kDa protein composed of 687 amino acid residues. The protein plays a significant role in numerous biological processes, including protein binding, apoptosis, wound healing, immune response, calcium signaling, and cellular differentiation^1–4^. The involvement of tTG in many biological processes leads to the protein being a potential therapeutic target for drug development in diseases such as renal fibrosis, Alzheimer’s disease, and celiac sprue^5–8^.

Tissue transglutaminase has gained significant interest as a drug target due to its central role in the molecular mechanism underlying celiac sprue. Individuals suffering from celiac disease, gluten proteins are broken down into oligopeptides in the small intestine. These peptides, characterized by their high glutamine content, interact with tTG, leading to their deamidation or transamidation. The resulting modified peptides are then presented to immune cells. Recognized by antigen-presenting cells as bacterial proteins, these deamidated peptides trigger an autoimmune inflammatory response in celiac disease patients. This response leads to the necrosis of small intestine cells and the production of antibodies against gluten proteins, tTG, and the tTG-peptide complex^9^. Although there may be no discernible difference in tTG structure between individuals with and without celiac disease, targeting tTG offers a potential therapeutic approach to mitigate the pathology of celiac disease^10^.

The tTG protein exists in two primary stable conformations: “open” and “closed”, depending on external conditions, such as pH, calcium concentration or presence of guanine nucleotides^11^. These conformations differ in functions, depending on their localization inside and outside of the cell, and although both configurations of tTG are usually found in the monomeric state, several studies have demonstrated that the open conformation of transglutaminase is also capable of dimerization^12^. While the closed tTG form acts as a part of G-protein and is not involved in the molecular mechanism of celiac sprue, the open form catalyzes either deamidation or transamidation of other proteins, playing a key role in the gluten peptide modification during the disease development^13,14^. As a result of tTG catalysis, ammonia is relieved, and the substrate-protein complex forms a thioester bond between cysteine residue of the active site and the substrate^15^. Although the substrate preference mechanism is still unclear for the tTG, there are specific motifs in peptides that showed high reactivity with the protein^16,17^.

The catalytic site of tissue transglutaminase encompasses multiple amino acids, each with extensively investigated functions. The nucleophilic cysteine (C277) plays a key part, attacking the substrate glutamine’s carboxamide group. This cysteine’s partial deprotonation is facilitated by a nearby histidine (H335) and an aspartate (D358) through a charge relay system. The active site is shielded by hydrophobic residues and located in a channel approached from opposite directions. W241 stabilizes intermediates of the protein-substrate complex through hydrogen bonding, while H305 and E363 residues facilitate the nucleophilic attack of the amine substrate at the acyl-enzyme intermediate^18^.

Modern tTG inhibitors are categorized based on their mechanism of action as competitive amine inhibitors, reversible and irreversible inhibitors^19^. Competitive amine inhibitors compete with natural amine substrates but exhibit low specificity under biological conditions. Reversible inhibitors predominantly act as allosteric inhibitors, mimicking GTP and inducing tTG into a closed conformation. Irreversible inhibitors form a complex with the enzyme, obstructing substrate access to the active site, predominantly binding with C277^20,21^. In complex with an irreversible inhibitor, tTG is locked in its open conformation and GTP binding is abolished^22^. Among the tTG inhibitors, peptidomimetic irreversible inhibitors have achieved significant strides, with one of their representatives, ZED1227, successfully completing Phase 2a clinical trials for celiac disease, thus demonstrating its safety and validating tTG as a viable drug target^23,24^. Compounds tested as tTG inhibitors at the moment of this study, according to the ChEMBL database (with up to 500 Standard Value), are listed in Table S1.1. Notably, previous in silico studies of tTG inhibitors relied on unverified molecular docking, therefore providing imprecise binding affinity data^25,26^.

The active site of the enzyme features an atypical saddle-like structure, which complicates the structure based drug design compared to the cases of well defined mostly hydrophobic sites^27^. At the moment of this study, the active site of tTG was known to possess four cavities available for inhibitor binding, therefore previous design of ligands targeting tTG relied on incomplete structural information about the enzyme.

We obtained a complete tTG model which will have a significant impact on the inhibitor development considering new structural insights about the active site architecture. The model reveals an additional loop that interacts with inhibitors in the active site and is absent in all available high-resolution models of tTG. This loop contains additional amino acid residues significantly affecting ligand binding affinity, which must be taken into account when performing molecular docking procedures.

We assembled a library of potential tTG inhibitors using a combination of known ChEMBL tTG ligands clustering and the search for analogous chemical scaffolds. We also calculated the binding affinity of the representative molecules from each of the chemical scaffolds by the developed and verified molecular docking procedure based on the complete tTG model. The assembled library expands the chemical space of tTG inhibitors, revealing eleven novel molecular scaffolds with outstanding binding affinity in comparison with the known one, and together with full structural information on tTG active site provides valid molecular docking results. Thus, our findings shed light on molecular mechanisms of ligand recognition by tTG, and open new directions for the design of selective and potent inhibitors.

## Results

### Analysis of tTG available structures

**Figure 1:**
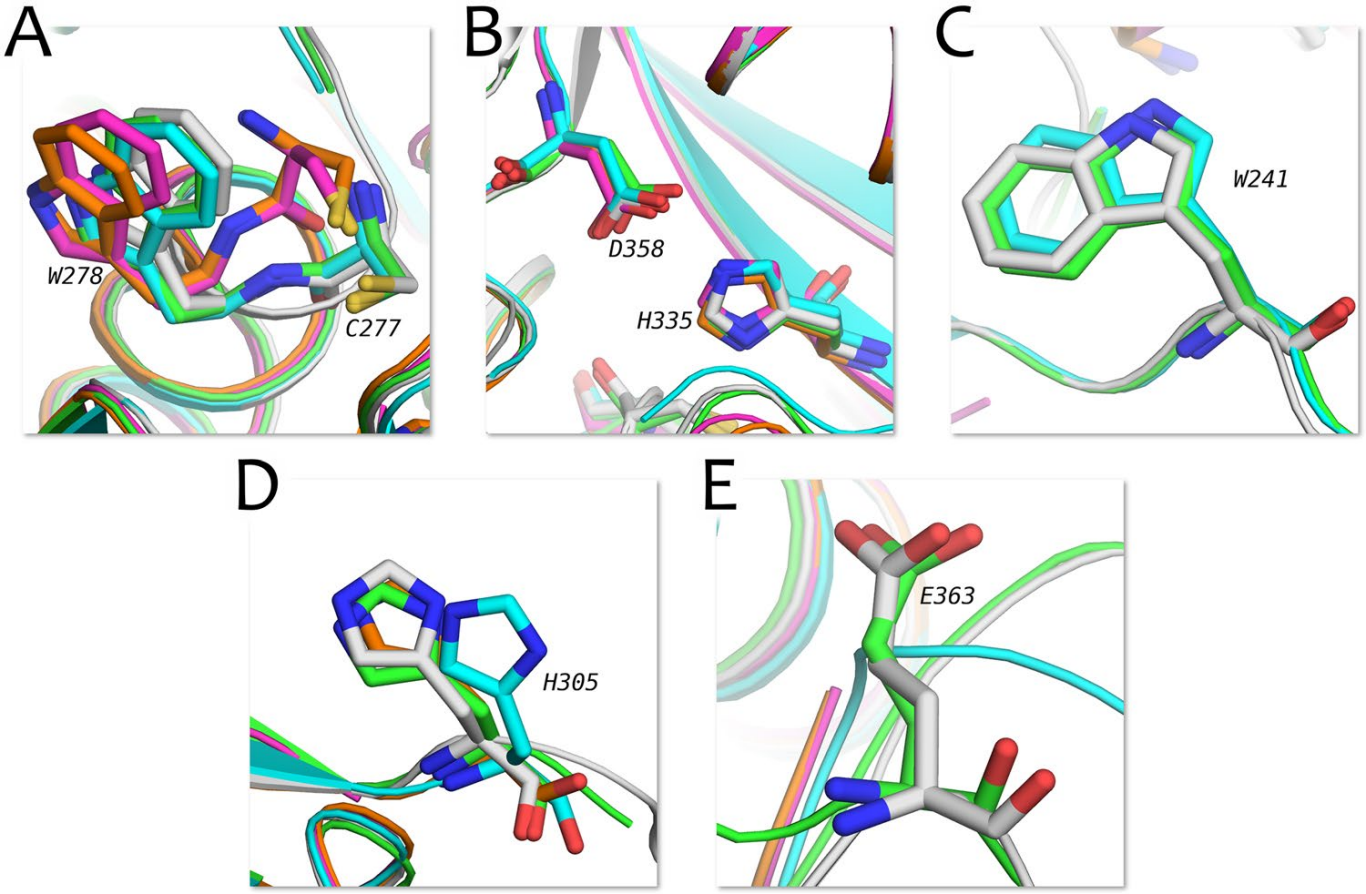
Comparison of amino acid residues taking part in the catalyzation of deamidation and transamidation between different PDB structures. 2q3z is shown in green, 3s3j in cyan, 3s3p in magenta, 3s3s in orange; the ColabFold-predicted structure is shown in light gray.

For computer simulations of the interaction between tissue transglutaminase and its inhibitors, a reliable and detailed model of the open conformation of the target protein is required. The high-resolution structures from the Protein Data Bank (for the open conformation of tTG: 2q3z, 3s3j, 3s3p, 3s3s) can serve as such a model^28^. We compared these structures to each other, paying special attention to the amino acid residues critical for the deamidation/transamidation process (Fig. 1).

**Figure 2:**
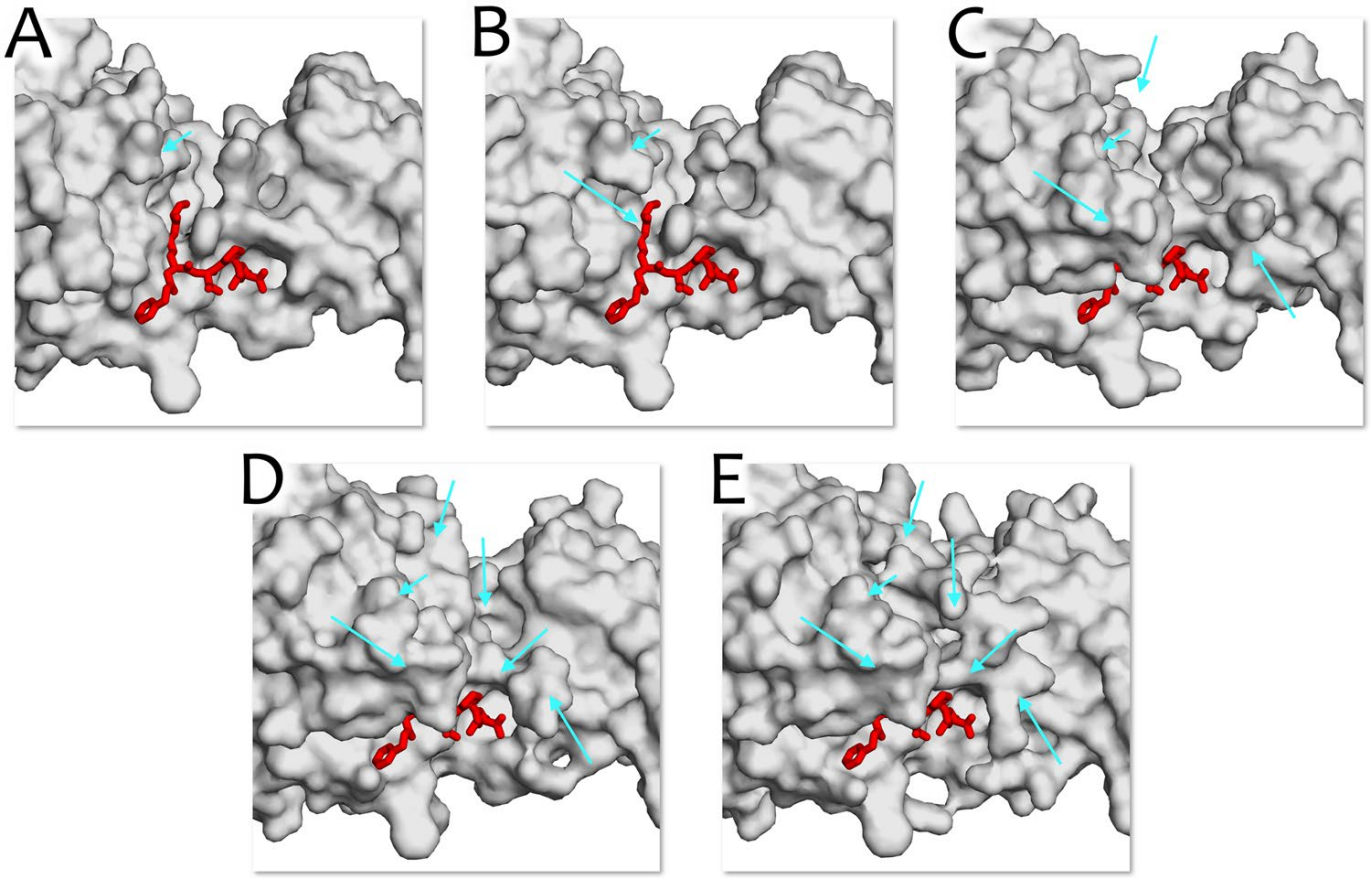
Comparison of the active site resolutions between different PDB structures. Ligand from 2q3z structure indicates the position of the active site and is shown in red. Significant differences are pointed at with cyan arrows. (**A**) tTG structure from 3s3s. (**B**) tTG structure from 3s3p. (**C**). tTG structure from 3s3j. (**D**) tTG structure from 2q3z. (**E**) The ColabFold-predicted tTG structure.

The positions of residues are close enough to each other between different high-resolution structures, and the protein backbone of tTG also remains consistent. This suggests limited mobility of the protein in this conformation, characterizing it as a convenient target for structure-based drug design. However, when comparing the known overall protein structures, significant differences can be noticed, which might influence the modeling results (Fig. 2). Since the structures obtained from PDB did not show the full protein’s integrity, the overall architecture of tTG protein differs as well as the active site structure, resulting in significant changes in the scenario of substrate-protein interaction for binding process simulations. The most relevant protein structure of the presented is from PDB:2q3z, where the loop forming the catalytic pocket has the most complete description. Even for this structure, part of the loop (residues E319-K327) forming the binding site is missing, which may hamper obtaining meaningful results of the structural based studies.

We applied a homology modeling method to derive a unified open conformation of the tissue transglutaminase (tTG) protein^29^. We compared four open conformation structures of tTG from the PDB to select the most homologous structure for modeling using Modeller v. 10.4 software^30^. Based on the comparative analysis, the 2q3z structure was chosen for homology modeling, resulting in the prediction of the complete tTG protein structure. However, upon closer examination of the predicted active site, a reduction in the binding surface can be observed, which might result in poorer docking outcomes.

An alternative method for modeling the full protein structure of tTG from its amino acid sequence is AlphaFold-based prediction. The closed conformation of the protein has already been successfully predicted using AlphaFold (ID: AF-P21980-F1). Modeling of tTG was done using the local version of ColabFold v.1.5.3 with the following parameters: num-recycle = 3, num-seeds = 10, tTG structure from PDB: 2q3z was used as a custom template, custom input msa comprising 36 sequences (including target sequence)^31–33^. MSA was performed in Unipro UGENE using Clustal Omega and was subsequently modified (amino acids matching from Q276 to C336 in the target sequence were changed to alanine to bias the prediction toward a desired conformational state)^34–37^.

As shown in Fig. 1, all amino acid residues critical in deamidation and transamidation reactions of the predicted tTG structure align well with those of the available PDB structures (PDB file of the structure can be found in Supplementary Materials). Furthermore, the derived structure (Fig. 2E) is the most complete and can provide the most precise insight into the actual protein–ligand interaction. Ultimately, the ColabFold-predicted protein structure proves to be the best target for the in silico development of tissue transglutaminase inhibitors.

### The architecture of the tTG active site

**Figure 3:**
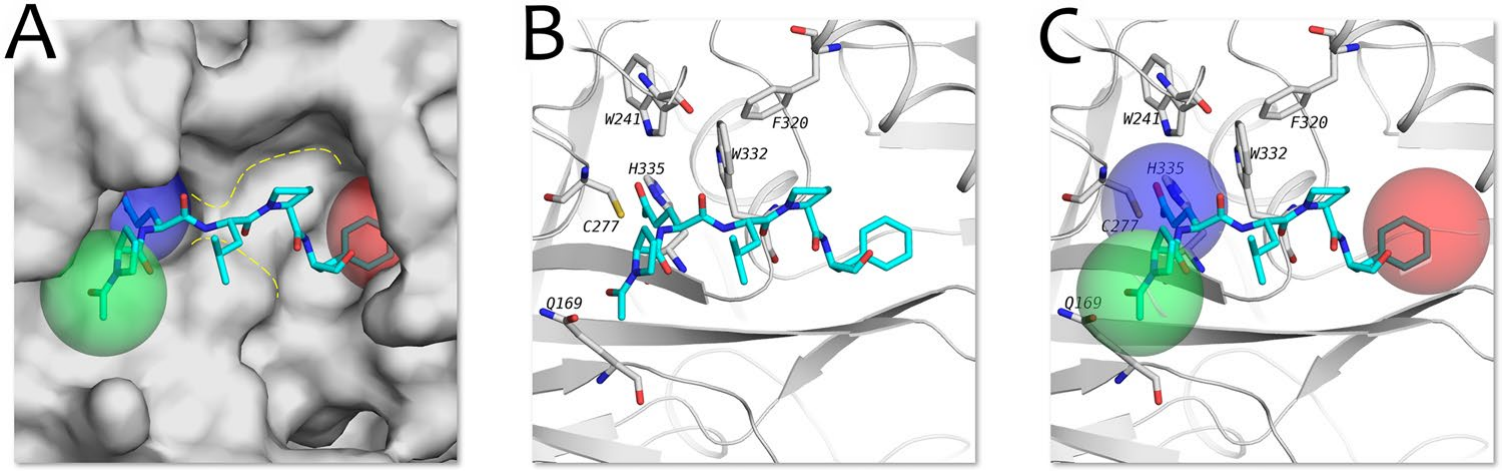
The active site of tTG. (**A**) Three cavities are schematically shown in green, blue and red spheres. Saddle-like structure is shown with the yellow dotted line. (**B**) Amino acid residues comprising cavities of the active site. (**C**) Comparison of cavities and amino acid residues. It is clear that the catalyzing cavity is shown as the blue sphere, with C277 residue inside of it.

A comprehension of the key features of the binding site of the open conformation of tTG is necessary to guide binding mode analysis and subsequent rational design (Fig. 3). The site predominantly resides on the protein surface and possesses a saddle-like shape, which poses challenges for virtual inhibitor design. Particularly, such architecture of the active site assumes the presence of a certain angle in the ligand core structure. For example, it can be reached by adding proline residue to the peptidomimetic inhibitors composition.

Based on the obtained structure, three cavities within the active site can be delineated, as referenced in Fig. 3A (marked schematically by coloured spheres). To achieve the surface area values significant to form reliable interactions with the active site, the ligand must occupy at least two of these three cavities. The biggest cavity (shown in red) is a wide pocket that can fit larger parts of the ligand such as aromatic groups. The cavity shown in green is located on the surface of tTG active site, and the cavity containing C277 residue (shown in blue) is a narrow deep pocket.

**Figure 4:**
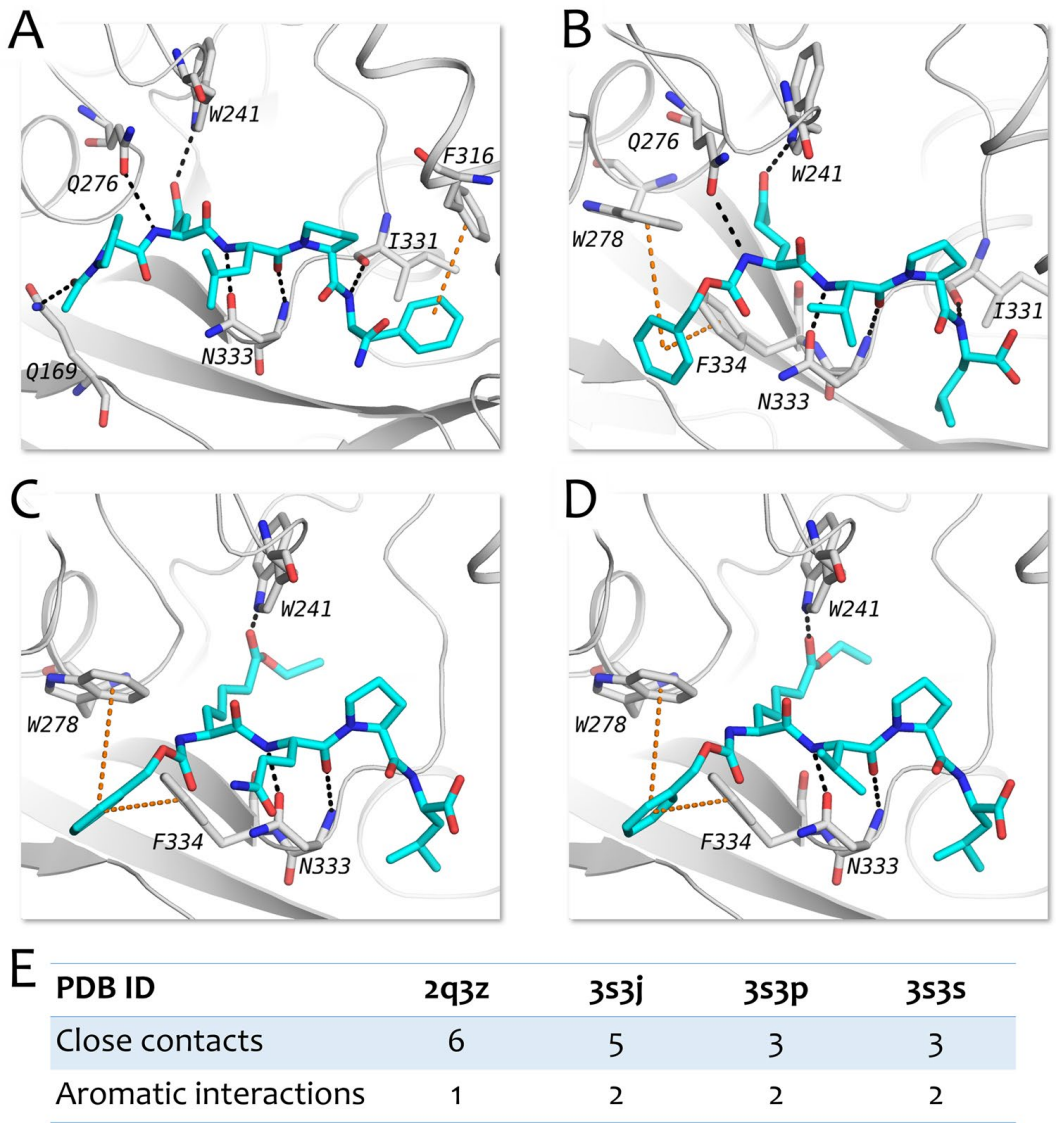
Interactions between ligands and the tTG active site. Close contacts are shown with black dotted lines, aromatic interactions are shown with orange dotted lines. (**A**) Ligand from 2q3z structure. (**B**) Ligand from 3s3j structure. (**C**) Ligand from 3s3p structure. (**D**) Ligand from 3s3s structure. (**E**) Number of close contacts and aromatic interactions calculated for each of the ligands.

Each of the accessible open-conformation protein structures in the Protein Data Bank (PDB) harbors an irreversible deamidated peptide-like inhibitor within its active site so that tTG stays in the open state. To elucidate the interactions between the amino acid residues of the active site and the ligand, close contacts (such as hydrogen bondings) and aromatic interactions (pi stacking, parallel displaced stacking, T-shaped stacking) were quantified in each of the four structures. The results of these calculations are shown in Fig. 4.

It is noteworthy that all compounds in the target-ligand complexes listed in the table exhibited similar characteristics (peptidomimetic structure containing 3 amino acid residues, size of compounds, ability to occupy all three of the tTG binding site cavities) both among themselves and when compared to the most successful commercial irreversible inhibitors of tissue transglutaminase (tTG). We also noticed that at the top of the binding site saddle, where the surface area of the ligand-site contact is lowered, all of the considered peptidomimetic inhibitors form two hydrogen bonds with N333 residue, compensating for the missing binding affinity and forming directed interactions. Finally, we observed the significance of an addition to the structure of the active site of the F320 residue, closing the gap existing in all open-state PDB structures of tTG.

**Figure 5:**
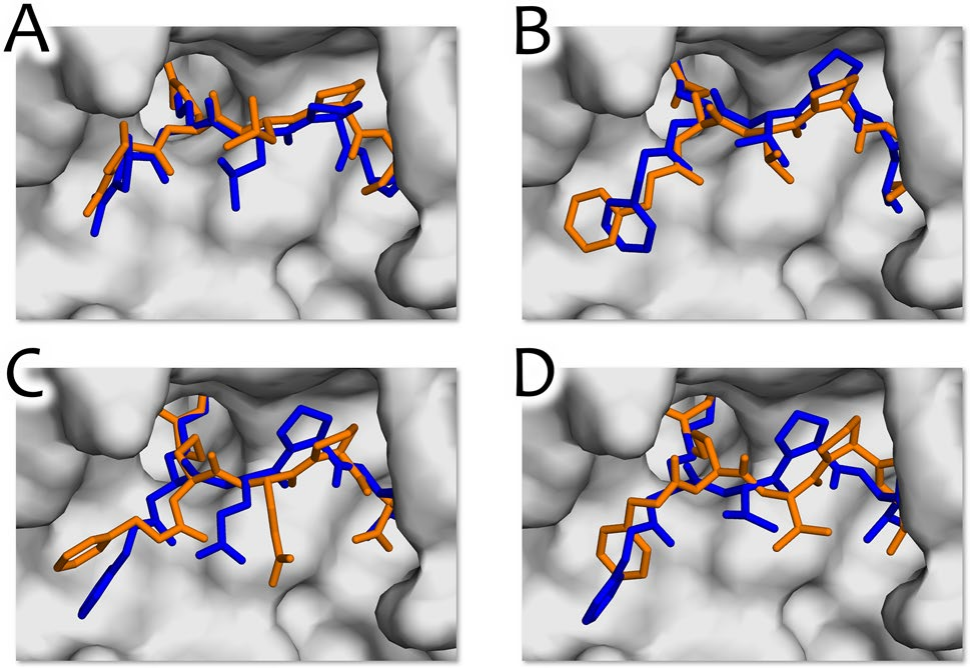
Molecular docking results in comparison to the original position of the ligand. The original position is shown in blue, the predicted position is shown in orange. (**A**). Cross-docking of 2q3z ligand to the tTG active site with the flexible Q169 residue. (**B**) Cross-docking of 3s3j ligand to the tTG active site with the flexible Q169 residue. (**C**) Cross-docking of 3s3p ligand to the tTG active site with the flexible Q169 residue. (**D**) Cross-docking of 3s3s ligand to the tTG active site with the flexible Q169 residue.

In order to assess binding affinity, molecular docking was conducted using the Gnina docking software^38,39^. The tTG structure obtained earlier with the AlphaFold-based algorithm was selected as the docking target. For validation of this target, we performed cross-docking of the ligand from 2q3z, 3s3j, 3s3p and 3s3s (Fig. 5). We optimized the docking outcomes by removing sulfur (which represented the thioester bond between C277 residue and substrate) from the active site of tTG. Additionally, flexible docking was done with amino acid residues W332, H335, W241, forming a cavity with catalyzing C277, and Q169, which forms another cavity (Fig. 3, in green), chosen based on the positions they could potentially occupy to enhance the binding affinity between the protein and ligand.

During the docking process, several issues were identified. Removing the excess sulfur from the active site accurately predicted the position of ligand 2q3z in the active site; however, it did not affect the negative results of the other dockings. Flexible docking of amino acid residues W241 and H335 enabled them to block access to the cavity with C277, adversely affecting the docking outcomes. Conversely, flexible docking of amino acid residue Q169 enhanced binding energy and led to precise ligand cross-docking results. However, the docking is inaccurate while predicting 3s3s and 3s3p ligand positions. We assume that the reason behind such results is that the part of both ligands responsible for covalent inhibition of C277 residue is too long and therefore does not fit into the cavity. Another reason for inaccurate docking results can lie in the excessive flexibility of peptidomimetic ligands: for instance, the ligand from PDB:3s3s has 20 rotatable links which makes it hard to enumerate all possible conformations.

In order to validate our assumptions, we performed 100 ns of molecular dynamics of our complete open-state tTG structure in apo mode and in complex with the ligand from 2q3z (position of which was precisely predicted by molecular docking) and with the ligand from 3s3s (docking position of which was less accurate) using Gromacs v.2022-6^45–47^. Open conformation of the protein remains consistent (apo mode RMSD, Fig. S1) and both ligands retain their position in the active site during the 100 ns simulation (ligand RMSD, Fig. S1). The active site itself is partially rearranged to gain more structural stability of the complex, which can also explain the difference between experimental positions of ligands and positions obtained from docking simulations.

Overall, the optimal molecular docking input parameters in the case of tissue transglutaminase have been estimated. Our ColabFold-predicted protein structure enables precise cross-docking of ligands, indicating its accuracy and the potential for structure-based drug design. Sulfur from the covalent binding of ligand and protein should be removed from the cavity, the flexible docking should be performed using Q169 as a flexible residue, and the exhaustiveness should be at least 32 to reach the most precise results.

### A library of new ligand-based and structure-based tTG-targeted small molecules

For the purpose of discerning which ligand in the known chemical space might exhibit optimal binding while remaining distinctive, we obtained a list of chemical compounds from the ChEMBL database that have been previously evaluated for binding to tTG and filtered based on their IC50 values. In result, a compilation of 169 compounds with known structural formulas and an IC50 < 500 nM was obtained.

In order to streamline the time-intensive process of selecting ligands, a clustering approach (Butina clustering) was employed based on the so-called Morgan circular fingerprints (presence of specific substructures) of radius 3 represented with 2048 bits, utilizing Tanimoto similarity measures^40,41^. We identified ten major clusters and chose one representative compound from each of the clusters (Table S1.2). These representatives were subsequently employed for docking procedures and searching for analogous molecules in the known chemical space.

In our search for similar substructures within the ChemRar database, a library of 2066 molecules potentially inhibiting tTG was assembled (Table S1.3). To discern the most prevalent patterns in this compound selection, we generated Bemis-Murcko scaffolds (BMS) of the original 169 compounds in ChEMBL and in the ChemRar compound library using the Rdkit library toolkit in Python^42^.

**Figure 6:**
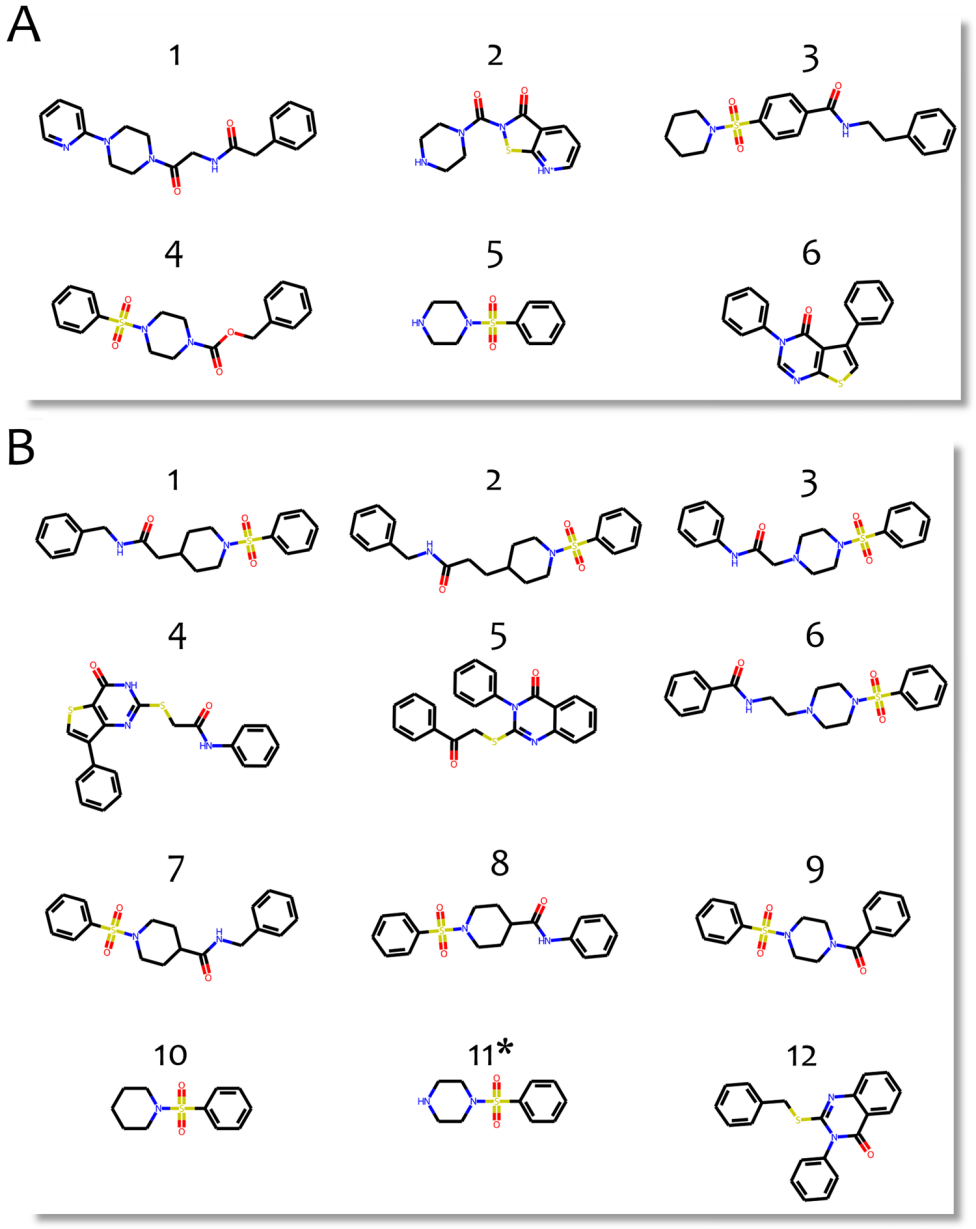
(**A**) Most frequent scaffolds from the ChEMBL dataset of tTG ligands. (**B**) Most frequent scaffolds from the assembled tTG-targeted library. The scaffolds identical between two datasets are indicated by an asterisk.

In order to determine which scaffolds were most commonly present in both datasets, we established an optimal threshold for a “sufficient” frequency of compound occurrences within a scaffold (set at 5 for ChEMBL compounds and 20 for ChemRar library compounds, respectively). This process yielded 6 scaffolds of experimentally verified compounds in ChEMBL and 12 scaffolds in the assembled library (Fig. 6). One scaffold was identical in both groups (scaffold 5 in the ChEMBL dataset and scaffold 11 in the ChemRar dataset), while the remaining scaffolds represented a different chemical space. The scaffold contains the sulfonic fragment providing the same angle for the core structure of small molecule ligands that is reached by proline residue in the peptidomimetic inhibitors.

We performed molecular docking of ten representative compounds from ChEMBL compound clustering using Gnina software with the ColabFold-predicted protein structure and the flexible residue Q169 to enhance binding affinity and precision of the docking process. To compare docking binding free energy results with experimental data from ChEMBL, we used the formula that relates binding free energy to the dissociation constant:

1 $$\Delta G = RTln\left( {\frac{{K_{d} }}{c}} \right), \quad c = 1 M$$

where dG is molar Gibbs free energy, Kd is dissociation constant, R is ideal gas constant, T is temperature and c is the standard reference concentration. All calculations are listed in Table S1.2. Though we observed no strong correlation between experimental ChEMBL data and modeling results, all obtained values differ by no more than 2 kcal/mol which can be regained by counting close contacts’ additional energy. The only compound with significant differences between experimental and modeling results is CHEMBL3423197, a compound with a relatively small size which explains lower energy of binding affinity.

Finally, to assess binding efficacy and develop a series within the tTG targeted library, five diverse compounds were selected by visual inspection for each of the 12 frequently occurring scaffolds. Utilizing our prior knowledge of ligand docking in the open conformation of tTG, we modeled the binding of each selected compound to the ColabFold-predicted protein structure (Table S1.2).

**Figure 7:**
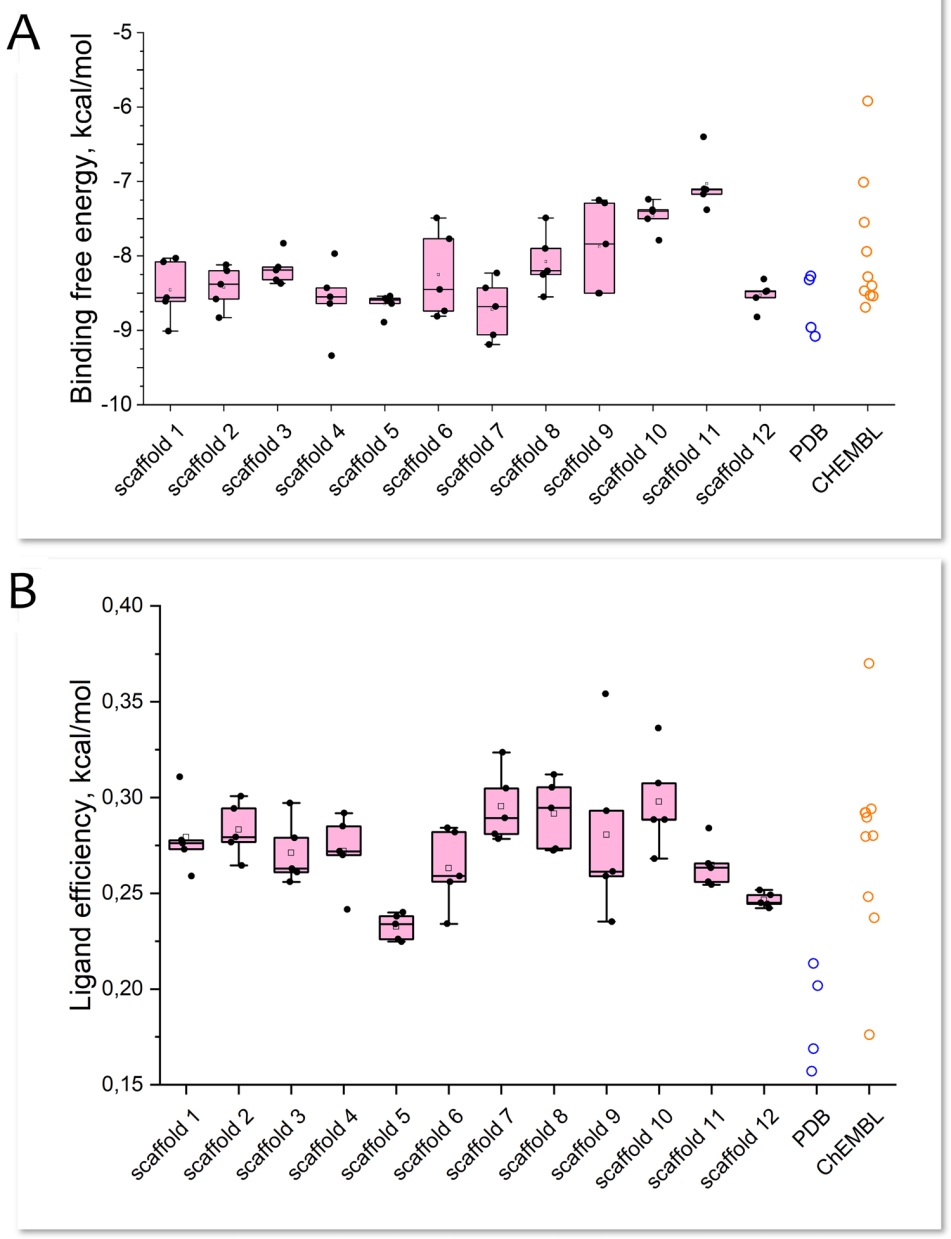
(**A**) Binding free energy of compounds from tTG-targeted library obtained by docking, 5 compounds per scaffold. Binding free energy of peptidomimetic compounds from PDB tTG structures obtained by docking is shown in blue, binding free energy of ChEMBL compounds is shown in orange. Dots outside boxplot whiskers are considered as outliers. (**B**) Ligand efficiency of compounds from tTG-targeted library, 5 compounds per scaffold. Binding efficiency of peptidomimetic compounds from PDB tTG structures obtained by docking is shown in blue, binding efficiency of ChEMBL compounds is shown in orange. Dots outside boxplot whiskers are considered as outliers.

The results of the molecular docking of small molecules were compared in terms of binding free energy both amongst themselves and with previously docked peptidomimetic inhibitors from the described PDB structures (Fig. 7). Notably, the closest energy values with the least scattering were observed for compounds with scaffolds 3–5 and 10–12, indicating a comparatively higher accuracy in the prediction for compounds with a similar core structure. Furthermore, scaffolds 4, 5, and 12 appeared to be more favorable for binding with tTG, whereas scaffolds 10 and 11 are less advantageous. The scaffold 11 is the only scaffold present in both ChEMBL and our tTG-targeted libraries; therefore, novel selected compounds from the library exhibit higher binding affinity to the enzyme than the already known compounds.

Peptidomimetic inhibitors showed one of the most favorable energy levels even with an inaccurate prediction of the ligand position (Fig. 5C,D). However, in terms of ligand efficiency (binding free energy per heavy atom), peptidomimetic ligands do not expectedly demonstrate a favorable profile compared to other compound groups. The compounds with scaffolds 7, 8 and 10, on the other hand, exhibit the highest binding efficiency.

In our analysis, we also identified most notable ligand outliers from the tTG targeted library (those deviating by more than 1 kcal/mol from other compounds in their group in terms of binding affinity, and more than 0.5 kcal/mol in terms of ligand efficiency). Compound with molecular scaffold 4, already one of the best groups of molecules in terms of affinity to tTG active site, shows exceptional binding free energy and therefore is of further research interest. Compound with molecular scaffold 9 has outstanding ligand efficiency, even though scaffold 9 itself is not one of the best ones in terms of affinity, and can be modified to the effective tTG inhibitor. Overall it can be stated that the promising scaffold-defined series of potential tTG inhibitors have been identified for further detailed analysis.

## Discussion

Tissue transglutaminase has been clinically validated as a promising drug target for a number of disorders, e.g. celiac disease^23^. However, the molecular mechanisms underlying the protein inhibition are yet to be thoroughly explored. tTG active site with its saddle-like structure can bind molecules up to 800 Da, and a ligand–protein interaction directly depends on the number of cavities in the active site architecture, as well as the number of close contacts and aromatic interactions. The analysis of the known binders shows that the kink shape of ligands ensured by the proline residue of peptide-like ligands and by sulfonic group in small molecule ligands is likely a crucial factor for favorable usage in case of saddle-like architecture of the binding site to enhance affinity of the ligands. In case of tTG targeting, ligand binding affinity might be strengthened by hydrogen bonding with the N333 amino acid backbone and its side chain group within the saddle-like region of the binding site. Subsequently, most of the revealed Bemis-Murcko scaffolds comprising our tTG-targeted library contain sulfonamide groups fulfilling the above requirements.

Contemporary methods in protein 3D-structure modeling have profoundly augmented our comprehension of molecular mechanisms of protein functioning which are pivotal for drug design. In this work, we successfully obtained the AlphaFold-predicted complete model of tTG in the open conformation. Importantly, to maintain such prediction efficacy of the protein conformation in some cases such as tTG, we used specific templates, as well as alanine replacement of amino acid residues in the active site. This signifies a next step in modeling protein flexibility and adaptability, which is crucial for studying the dynamics of proteins.

When considering the molecular docking of compounds, the obtained complete model of tissue transglutaminase has shown the most precise and relevant cross-docking results. This structure also resolves the loop forming the catalytic pocket in open conformation of tTG. Such a conformation accommodates the critical regions of trial ligands more effectively, thereby providing a more realistic and practical framework for drug design. Accurate modeling of these structural features not only enhances the reliability of the docking simulations but also opens avenues for designing more effective and targeted therapeutic agents.

Regarding the docking process itself, the Gnina software accurately predicted the most probable position for tTG ligands by combining Autodock Vina software and convolutional neural network models. For more accurate protein–ligand binding simulation results for non-covalently binding inhibitors, it is necessary to remove sulfur associated with covalent inhibition from the active site of tTG. Additionally, flexible docking of the Q169 residue significantly enhances both docking precision and the resulting binding free energy. However, the inclusion of other seemingly related residues to the flexible part of docking did not improve our results. Thus, we designed a docking procedure to accurately predict both small molecule and polypeptide binding modes without excessive computations.

We suggest that the tTG C277-containing cavity is still not sufficiently exposed for modeling binding with large compounds based on the following observations. The first is an increased affinity obtained by molecular docking when the ligand occupies the gorge of this cavity in comparison to decreased affinity of other ligands which include the same scaffold and do not occupy the cavity. The second reason is that during the cross-docking of ligands from PDB: 3s3s and PDB: 3s3p, they were not predicted precisely due to the size of the covalent binding part which is supposed to bind to C277. Flexible docking of amino acids forming this cavity leads to its “blockage”, subsequently preventing ligand entry. Furthermore, the protein structure obtained by ColabFold has the same size of C277-containing cavity as that in available high-resolution PDB structures of tTG, indicating that molecular dynamics, perhaps in a restrained ligand–protein form, remains the only viable method for identifying even more accurate and reliable active site conformations for future research.

A reasonable option in the case of reliable protein structure might be to combine the obtained complete protein structure together with molecular dynamics modeling to select relevant conformations (either apo or ligand–protein complex forms) with their subsequent clusterization. The selected protein conformations then can be used within the ensemble docking approach to screen libraries of potential tTG inhibitorz^43,44^. We briefly checked this hypothesis by performing molecular dynamics of our complete tTG structure in apo mode and in a complex with 2 different peptidomimetic ligands (Fig. S1). As a result of MD simulations in apo mode, several amino acid residues in the active site slightly changed their positions, exposing the catalytic C277-containing cavity (which corresponds to the assumption mentioned above on the docking procedure of the large compounds). However, the backbone of the protein remained in the same position during the simulation. As for MD simulations of the two ligand–protein complexes, the protein backbone also remains consistent, as well as all of the secondary structures. At the same time, the catalytic pocket deepens in both cases, allowing the ligand to move further into the active site (in the case of ligand from PDB: 3s3s, bringing the predicted ligand position closer to the experimentally obtained position). We also noticed that during the MD simulations, both ligands occupied all three defined cavities (Fig. 3), and conserved all close contacts and aromatic interactions with the active site (Fig. 4), showing the consistency with the ligand binding modes.

We compared the key amino acid residues (Fig. 1) positions before and after the MD simulations and found two significant differences. Firstly, W241 slightly changes its position and rotates, which results in the more accessible shape of the C277-containing cavity. In addition, E363 (the most distant key amino acid residue from the active site) also significantly changes its former position because of the movements of the flexible loop where the residue is located. The residue’s position after the MD simulations, however, remains consistent between all of the simulations; therefore, we suggest that the change in position of E363 relates to its function in the ligand deprotonation events, which requires conformational changes in order to bring E363 closer to H305. As for all of the other key amino acid residues, their position does not change during the whole simulation process, which indicates the stability of the catalytic part of the active site in apo mode as well as in complex with different ligands.

Therefore, our complete open-state tissue transglutaminase structure predicted by AlphaFold-based algorithm is validated using both molecular docking and molecular dynamics simulations. It is shown that MD simulation using the protein structure successfully predicts ligand binding modes, so the structure can be used in further studies of tTG inhibitors. It is also shown that the obtained structure can be potentially used to explore the conformational changes of the protein depending on ligands as well as external conditions.

Our research expands the chemical space of compounds potentially targeting tTG and systematizes these compounds in the new tTG-targeted library. Among the compounds proposed for further experimental testing, we have highlighted twelve Bemis-Murcko scaffolds, which can be used for subsequent structure–activity relationship analysis, with three scaffolds predicted as the most promising (Fig. 6). Additionally, we showed that whereas docking results based on our complete structure of tTG indicate that peptidomimetic inhibitors are favorable in terms of the binding free energy, the binding efficiency is comparable across all twelve scaffolds (ranging from 0.2 to 0.35 kcal/mol, Fig. 7). This highlights the potential for affinity optimization towards successful finding of hits in further research. Finally, due to greater bioavailability, small molecules could become successful alternatives to the peptidomimetic inhibitors as drug molecules targeting tissue transglutaminase. All of this shapes the promising future experimental research^50^.

We acknowledge that although the study presents promising results from molecular modeling and docking simulations, there are inherent limitations and uncertainties associated with computational predictions and, without experimental validation, the accuracy and reliability of the computational findings remain uncertain. Therefore, the potential utility of the computational results should be experimentally validated to confirm the efficacy of the proposed tTG inhibitors.

## Materials and Methods

### AlphaFold-based prediction of the complete open-state structure of tTG

In order to predict the complete open-state structure of tTG, we performed modeling of the protein using the local version of ColabFold v.1.5.3 with the following parameters: num-recycle = 3, num-seeds = 10. The protein structure from PDB ID: 2q3z was used as a custom template. MSA was performed in Unipro UGENE using Clustal Omega, amino acids matching from Q276 to C336 in the target sequence were changed to alanine.

### Molecular docking of tTG ligands in the active site

Molecular docking of ligands was performed using Gnina v.1.0 software with the following parameters: obtained AlphaFold-predicted complete tTG structure as a receptor molecule, Q169 (A:169) as a flexible residue, exhaustiveness = 32^38,39^.

All ligands for cross-docking were prepared to produce PDBQT format using OpenBabel v.3.1.1^51^.

### Molecular dynamics of tTG ligands in the active site

Molecular dynamics was done using Gromacs v.2022-6 with CHARMM36 force field^45–47^. Each complex was solvated within a box of the transferable intermolecular potential with a three-points (TIP3P) water model. The systems were prepared for MD simulation: the apo form of tTG2, and two complexes of the tTG2 with ligands from PDB:2Q3Z and PDB:3S3S with initial positions obtained during the docking. The solvated and Na + supplemented systems were first energy minimized for 50,000 steps using Steepest Descent algorithm with positional restraint on the initial geometry. Then the NVT and NPT system equilibration were conducted for 100 ps each with preserving the restraints on protein and ligand positions, using target temperature of 300 K and temperature coupling parameter tau_t = 0.1 ps (V-rescale algorithm) as well pressure coupling parameter tau_p = 2 ps (Berendsen algorithm). Production MD runs were conducted with the same NPT settings and held for 100 ns for each system. LINCS constraints were applied, so that 2 fs time step was applied for integration.

### Assembling of the library of new potential tTG inhibitors

Ten active structures were identified in the ChEMBL database with the tTG IC50 records below 500 nM: CHEMBL2089386, CHEMBL2203473, CHEMBL2152099, CHEMBL180595, CHEMBL3891796, CHEMBL2089393, CHEMBL3423197,

CHEMBL2086539, CHEMBL3092841, and CHEMBL2086536. The most similar structures were extracted from the ChemRar compound database using ECFP (Morgan fingerprints in RDKit) of radius 3 and 2048 bits and Tanimoto similarity at the threshold of 0.3. The obtained compounds were filtered for PAINS^48^ and Ro5 filters^49^. The filtered compounds were subjected to clustering using Butina algorithm^40^ on top of the fingerprints described above, with the most populated 12 clusters (sharing the same scaffold) being used for further analysis as potential scaffolds forming a series. The latter is essential to increase the chances of the developability of an initial hit to leads and drugs.

The space geometries of the studied small molecule compounds were generated by DataWarrior v.05.05.00 software using option “Generate conformers” with the following input parameters: algorithm—random; low energy bias; forcefield—MMFF94s + ; maximum conformer count—1 per stereoisomer^52^.

### Supplementary Information

Supplementary Information 1.

Supplementary Figure 1.

Supplementary Tables.

## Data Availability

The authors declare that the data supporting the findings of this study are available within the paper and its Supplementary Material. Raw data that support the findings of this study are available from the corresponding author upon reasonable request.

## References

[1] Aeschlimann D, Mosher D, Paulsson M (1996). Tissue transglutaminase and factor XIII in cartilage and bone remodeling. Semin. Thromb. Hemost..

[2] Cho S-Y, Lee J-H, Bae H-D, Jeong EM, Jang G-Y, Kim C-W, Shin D-M, Jeon J-H, Kim I-G (2010). Transglutaminase 2 inhibits apoptosis induced by calciumoverload through down-regulation of Bax. Exp. Mol. Med..

[3] Tatsukawa H, Hitomi K (2021). Role of transglutaminase 2 in cell death, survival, and fibrosis. Cells.

[4] Tonoli E, Verduci I, Gabrielli M, Prada I, Forcaia G, Coveney C, Savoca MP, Boocock DJ, Sancini G, Mazzanti M, Verderio C, Verderio EAM (2022). Extracellular transglutaminase-2, nude or associated with astrocytic extracellular vesicles, modulates neuronal calcium homeostasis. Prog. Neurobiol..

[5] Shinoda Y, Tatsukawa H, Yonaga A, Wakita R, Takeuchi T, Tsuji T, Tanaka M, Suganami T, Hitomi K (2023). Tissue transglutaminase exacerbates renal fibrosis via alternative activation of monocyte-derived macrophages. Cell Death Dis..

[6] Wilhelmus MMM, Tonoli E, Coveney C, Boocock DJ, Jongenelen CAM, Brevé JJP, Verderio EAM, Drukarch B (2022). The transglutaminase-2 interactome in the APP23 mouse model of Alzheimer’s disease. Cells.

[7] Iversen R, Sollid LM (2015). Transglutaminase 2 and celiac disease. Transglutaminases.

[8] Fesus L, Piacentini M (2002). Transglutaminase 2: an enigmatic enzyme with diverse functions. Trends Biochem. Sci..

[9] Green PHR, Cellier C (2007). Celiac disease. New Engl. J. Med..

[10] Sulic A-M, Kurppa K, Rauhavirta T, Kaukinen K, Lindfors K (2014). Transglutaminase as a therapeutic target for celiac disease. Expert Opin. Therap. Targ..

[11] Katt WP, Antonyak MA, Cerione RA (2018). Opening up about tissue transglutaminase: When conformation matters more than enzymatic activity. Med. One.

[12] Kim N, Lee W-K, Lee S-H, Jin KS, Kim K-H, Lee Y, Song M, Kim S-Y (2016). Inter-molecular crosslinking activity is engendered by the dimeric form of transglutaminase 2. Amino Acids.

[13] Nurminskaya MV, Belkin AM (2012). Cellular functions of tissue transglutaminase. Int. Rev. Cell Mol. Biol..

[14] Pinkas DM, Strop P, Brunger AT, Khosla C (2007). Transglutaminase 2 undergoes a large conformational change upon activation. PLoS Biol..

[15] Savoca M, Tonoli E, Atobatele A, Verderio E (2018). Biocatalysis by transglutaminases: A review of biotechnological applications. Micromachines.

[16] Damnjanović J, Odake N, Fan J, Camagna M, Jia B, Kojima T, Nemoto N, Hitomi K, Nakano H (2022). Comprehensive analysis of transglutaminase substrate preference by cDNA display coupled with next-generation sequencing and bioinformatics. Sci. Rep..

[17] Sugimura Y, Hosono M, Wada F, Yoshimura T, Maki M, Hitomi K (2006). Screening for the preferred substrate sequence of transglutaminase using a phage-displayed peptide library. J. Biol. Chem..

[18] Demény MÁ, Korponay-Szabó I, Fésüs L (2015). Structure of transglutaminases: Unique features serve diverse functions. Transglutaminases.

[19] Siegel M, Khosla C (2007). Transglutaminase 2 inhibitors and their therapeutic role in disease states. Pharmacol. Therap..

[20] Keillor JW, Chabot N, Roy I, Mulani A, Leogane O, Pardin C (2011). Irreversible inhibitors of tissue transglutaminase. Adv. Enzymol. Relat. Areas Mol. Biol..

[21] Cundy NJ, Arciszewski J, Gates EWJ, Acton SL, Passley KD, Awoonor-Williams E, Boyd EK, Xu N, Pierson É, Fernandez-Ansieta C, Albert MR, McNeil NMR, Adhikary G, Eckert RL, Keillor JW (2023). Novel irreversible peptidic inhibitors of transglutaminase 2. RSC Med. Chem..

[22] Navals P, Rangaswamy AMM, Kasyanchyk P, Berezovski MV, Keillor JW (2024). Conformational modulation of tissue transglutaminase via active site thiol alkylating agents: Size does not matter. Biomolecules.

[23] Büchold C, Hils M, Gerlach U, Weber J, Pelzer C, Heil A, Aeschlimann D, Pasternack R (2022). Features of ZED1227: The first-in-class tissue transglutaminase inhibitor undergoing clinical evaluation for the treatment of celiac disease. Cells.

[24] Isola J, Mäki M, Hils M, Pasternack R, Viiri K, Dotsenko V, Montonen T, Zimmermann T, Mohrbacher R, Greinwald R, Schuppan D (2023). The oral transglutaminase 2 inhibitor ZED1227 accumulates in the villous enterocytes in celiac disease patients during gluten challenge and drug treatment. Int. J. Mol. Sci..

[25] Tadayon M, Garkani-Nejad Z (2018). Quantitative structure–activity relationship study using genetic algorithm–enhanced replacement method combined with molecular docking studies of isatin derivatives as inhibitors of human transglutaminase 2. J. Chin. Chem. Soc..

[26] Keillor JW (2015). Inhibition of Transglutaminase. Transglutaminases.

[27] Zheng X, Gan L, Wang E, Wang J (2012). Pocket-based drug design: Exploring pocket space. AAPS J..

[28] Berman HM (2000). The protein data bank. Nucleic Acids Res..

[29] Muhammed MT, Aki-Yalcin E (2018). Homology modeling in drug discovery: Overview, current applications, and future perspectives. Chem. Biol. Drug Design.

[30] Webb B, Sali A (2016). Comparative protein structure modeling using MODELLER. Curr. Protoc. Bioinf.

[31] Mirdita M, Schütze K, Moriwaki Y, Heo L, Ovchinnikov S, Steinegger M (2022). ColabFold: Making protein folding accessible to all. Nat. Methods.

[32] Jumper J, Evans R, Pritzel A, Green T, Figurnov M, Ronneberger O, Tunyasuvunakool K, Bates R, Žídek A, Potapenko A, Bridgland A, Meyer C, Kohl SAA, Ballard AJ, Cowie A, Romera-Paredes B, Nikolov S, Jain R, Adler J, Hassabis D (2021). Highly accurate protein structure prediction with AlphaFold. Nature.

[33] Evans R (2021). Protein complex prediction with AlphaFold-Multimer. bioRxiv.

[34] Okonechnikov K, Golosova O, Fursov M (2012). Unipro UGENE: A unified bioinformatics toolkit. Bioinformatics.

[35] Sievers F, Wilm A, Dineen D, Gibson TJ, Karplus K, Li W, Lopez R, McWilliam H, Remmert M, Söding J, Thompson JD, Higgins DG (2011). Fast, scalable generation of high-quality protein multiple sequence alignments using Clustal Omega. Mol. Syst. Biol..

[36] Sala D, Engelberger F, Mchaourab HS, Meiler J (2023). Modeling conformational states of proteins with AlphaFold. Curr. Opin. Struct. Biol..

[37] Stein RA, Mchaourab HS (2022). SPEACH_AF: Sampling protein ensembles and conformational heterogeneity with Alphafold2. PLOS Comput. Biol..

[38] McNutt AT, Francoeur P, Aggarwal R, Masuda T, Meli R, Ragoza M, Sunseri J, Koes DR (2021). GNINA 1.0: Molecular docking with deep learning. J. Cheminf..

[39] Ragoza M, Hochuli J, Idrobo E, Sunseri J, Koes DR (2017). Protein-Ligand scoring with convolutional neural networks. J. Chem. Inf. Model..

[40] Butina D (1999). Unsupervised data base clustering based on daylight’s fingerprint and Tanimoto similarity: A fast and automated way to cluster small and large data sets. J. Chem. Inf. Comput. Sci..

[41] Zhong S, Guan X (2023). Count-based Morgan fingerprint: A more efficient and interpretable molecular representation in developing machine learning-based predictive regression models for water contaminants’ activities and properties. Environ. Sci. Technol..

[42] RDKit: Open-source cheminformatics. https://www.rdkit.org10.5281/ZENODO.591637

[43] Amaro RE, Baudry J, Chodera J, Demir Ö, McCammon JA, Miao Y, Smith JC (2018). Ensemble docking in drug discovery. Biophys. J..

[44] Korb O, Olsson TSG, Bowden SJ, Hall RJ, Verdonk ML, Liebeschuetz JW, Cole JC (2012). Potential and limitations of ensemble docking. J. Chem. Inf. Model..

[45] Bauer, P., Hess, B., & Lindahl, E. (2023). GROMACS 2022.6 Manual. 10.5281/ZENODO.6103567

[46] Lemkul J (2019). From proteins to perturbed hamiltonians: A suite of tutorials for the GROMACS-2018 molecular simulation package [Article v1.0]. Living J. Comput. Mol. Sci..

[47] da Silva TU, de Pougy KC, Albuquerque MG, da Silva Lima CH, de Machado SP (2020). Development of parameters compatible with the CHARMM36 force field for [Fe_4_S_4_]^2+^ clusters and molecular dynamics simulations of adenosine-5’-phosphosulfate reductase in GROMACS 2019. J. Biomol. Struct. Dyn..

[48] Baell JB, Holloway GA (2010). New substructure filters for removal of pan assay interference compounds (PAINS) from screening libraries and for their exclusion in bioassays. J. Med. Chem..

[49] Lipinski CA, Lombardo F, Dominy BW, Feeney PJ (1997). Experimental and computational approaches to estimate solubility and permeability in drug discovery and development settings. Adv. Drug Deliv. Rev..

[50] Yakubov B, Chen L, Belkin AM, Zhang S, Chelladurai B, Zhang Z-Y, Matei D (2014). Small molecule inhibitors target the tissue transglutaminase and fibronectin interaction. PLoS ONE.

[51] O’Boyle NM, Banck M, James CA, Morley C, Vandermeersch T, Hutchison GR (2011). Open babel: An open chemical toolbox. J. Cheminf..

[52] Sander T, Freyss J, von Korff M, Rufener C (2015). DataWarrior: An open-source program for chemistry aware data visualization and analysis. J. Chem. Inf. Model..

