# Supplementary figures and images for: In silico studies of the open form of human tissue transglutaminase
Source: Sci Rep. 2024 Jul 10;14:15981. doi: 10.1038/s41598-024-66348-8 (PMC11236986; doi:10.1038/s41598-024-66348-8)

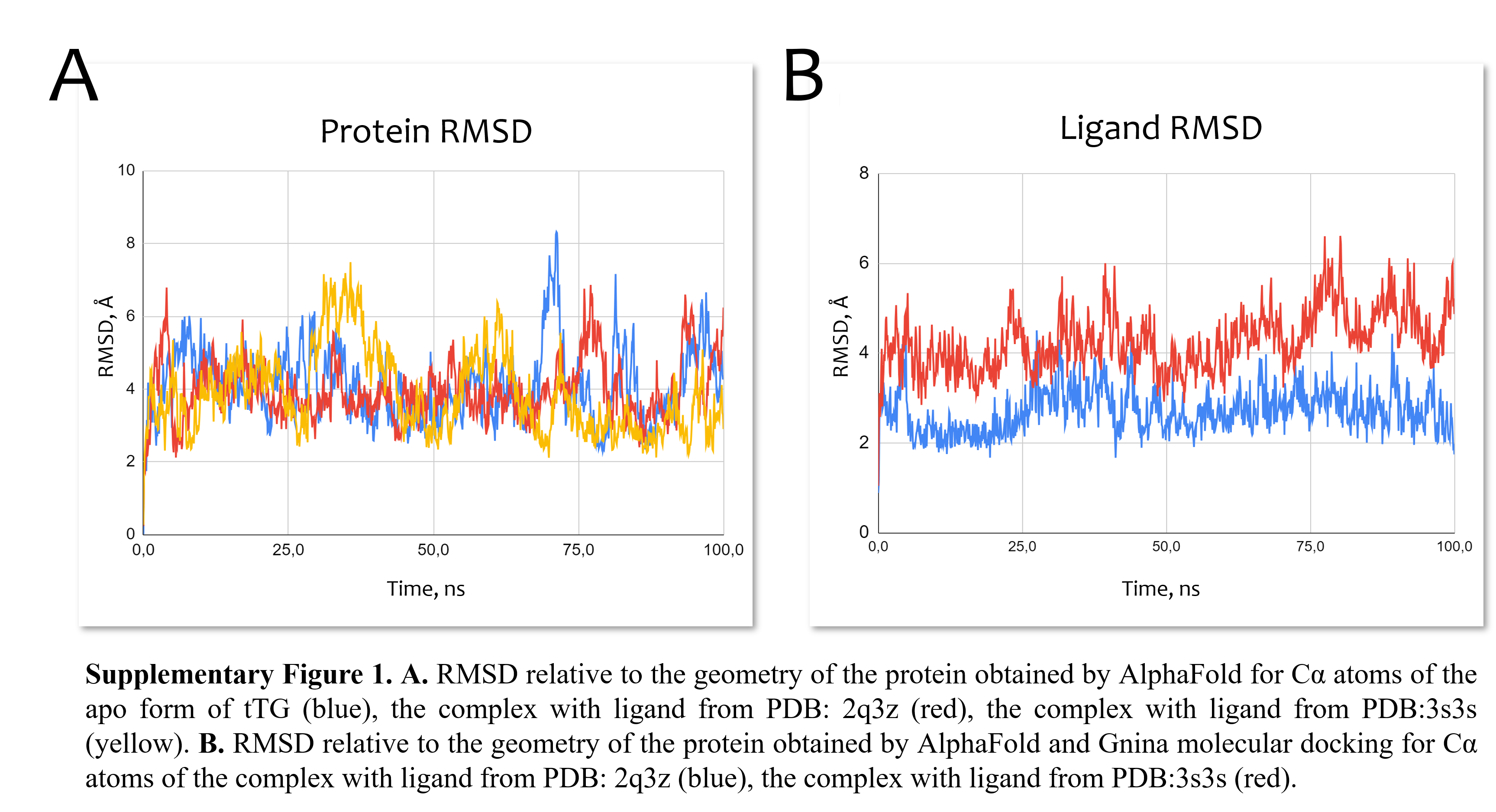

Supplement: Supplementary file 2 — Supplementary Figure 1. [file 41598_2024_66348_MOESM2_ESM.png]
